# Traditional Malian Solid Foods Made from Sorghum and Millet Have Markedly Slower Gastric Emptying than Rice, Potato, or Pasta

**DOI:** 10.3390/nu10020124

**Published:** 2018-01-26

**Authors:** Fatimata Cisse, Daniel P. Erickson, Anna M. R. Hayes, Antone R. Opekun, Buford L. Nichols, Bruce R. Hamaker

**Affiliations:** 1 Whistler Center for Carbohydrate Research, Department of Food Science, Purdue University, West Lafayette, IN 47907, USA; diallofati@gmail.com (F.C.); daniel.erickson@rd.nestle.com (D.P.E.); hayes100@purdue.edu (A.M.R.H.); 2 Institut d’Economie Rurale du Mali (IER), BP 258 Bamako, Mali; 3 Department of Pediatrics, Baylor College of Medicine, Houston, TX 77030, USA; aopekun@bcm.edu (A.R.O.); blnjr@sbcglobal.net (B.L.N.)

**Keywords:** Africa, staple foods, gastric emptying, satiety, sustained energy

## Abstract

From anecdotal evidence that traditional African sorghum and millet foods are filling and provide sustained energy, we hypothesized that gastric emptying rates of sorghum and millet foods are slow, particularly compared to non-traditional starchy foods (white rice, potato, wheat pasta). A human trial to study gastric emptying of staple foods eaten in Bamako, Mali was conducted using a carbon-13 (^13^C)-labelled octanoic acid breath test for gastric emptying, and subjective pre-test and satiety response questionnaires. Fourteen healthy volunteers in Bamako participated in a crossover design to test eight starchy staples. A second validation study was done one year later in Bamako with six volunteers to correct for endogenous ^13^C differences in the starches from different sources. In both trials, traditional sorghum and millet foods (thick porridges and millet couscous) had gastric half-emptying times about twice as long as rice, potato, or pasta (*p* < 0.0001). There were only minor changes due to the ^13^C correction. Pre-test assessment of millet couscous and rice ranked them as more filling and aligned well with postprandial hunger rankings, suggesting that a preconceived idea of rice being highly satiating may have influenced subjective satiety scoring. Traditional African sorghum and millet foods, whether viscous in the form of a thick porridge or as non-viscous couscous, had distinctly slow gastric emptying, in contrast to the faster emptying of non-traditional starchy foods, which are popular among West African urban consumers.

## 1. Introduction

Sorghum and millets constitute a main source of nutrition in diets of people living in the semi-arid regions of Africa. These crops are able to grow in droughty conditions and, since mostly disadvantaged people consume them, they are considered by the Food and Agriculture Organization of the United Nations (FAO) to be “crops for poor people” [1]. Traditional West African sorghum and millet foods are served in different solid forms (e.g., medium to very thick porridges, couscous, and larger particle size agglomerated products) as well as liquid forms (e.g., thin porridges with and without granules) [2]. Factors such as urbanization and improved economic status have prompted changes in the dietary habits of Africans, with a shifting away from such traditional foods to imported or Western foods [1,3,4,5,6,7,8]. This dietary trend in urban areas is termed the “nutrition transition”, and is thought to be a causative factor in the increasing prevalence of obesity and associated diseases (e.g., diabetes and cardiovascular disease) [6]. Similarly, high risks of obesity and type 2 diabetes were seen among sub-Saharan African populations living in Europe and in the urban environment of Ghana [3]. Rapid health deterioration and increases in obesity and chronic disease in sub-Saharan African migrants to Australia were attributed to changes in dietary habits away from traditional foods [9].

Anecdotal evidence in the region suggests that sorghum and millet foods are filling and provide extended energy after consumption. This is supported by in vitro and in vivo studies showing comparably slow starch digestion in these foods [10,11,12,13]. In sorghum, low starch digestibility was linked to a protein-starch interaction [14]. In part, the satiating property of sorghum and millet foods could be due to gastric emptying rate, which is a key regulator of macronutrient delivery to the body and is positively correlated to glycemic response [15,16]. It largely affects whether energy is delivered from a meal in a rapid or slow fashion. Several factors directly influence gastric emptying rate, including nutrient content, physical properties (e.g., viscosity, particle size, and texture), volume, and macronutrient properties.

Sorghum (low or tannin-free types) and millet are commonly consumed in West Africa as thick porridges, and for these foods their viscosity property could slow gastric emptying. Highly viscous foods are thick and resistant to flow; thus, their passage through the stomach may be longer in duration than less viscous foods. Viscous gums are known to reduce gastric emptying rate and postprandial glycemic response [17], and highly viscous foods have reduced gastric emptying rate and increased satiety [18,19,20,21]. Viscosity and volume are associated with satiety [22]. Yet, not all sorghum and millet West African foods are viscous porridges, such as couscous, and these types of foods are also associated with satiety and fullness. Additionally, sorghum and millet foods are known to have relatively low starch digestibility which might slow gastric emptying by reaching the ileum to trigger the ileal brake mechanism [13,23,24,25,26,27,28].

Here, we tested the hypothesis that traditional foods of the West Africa Sahel made from sorghum and millet, both viscous and not viscous in nature, have slow gastric emptying compared to non-traditional starchy foodstuffs (rice, potatoes, wheat pasta) that are commonly consumed today in urban areas, which would contribute to a satiety response. A broader aspect of this work is to understand whether sorghum and millet foods of West Africa have positive nutritional attributes, which could be used to promote these grains in urban areas to provide expanded markets for local smallholder farmers. 

## 2. Materials and Methods

### 2.1. Subject Selection

A human study was conducted in Bamako, Mali followed by a validation trial approximately one year later to correct for differences in background carbon-13 (^13^C) in different starchy food stuffs. Pilot data were used to determine the minimum sample size (0.05 level of significance, 0.5 h minimum detectable difference in means, 0.8 power, 0.147 h within subject standard deviation, *n* ≥ 4). Eligibility criteria were: males or females aged 20–50 years, normal body mass index (BMI, 18 kg/m^2^ ≤ BMI ≤ 25 kg/m^2^), not under any medication, no history of any gastrointestinal disease or surgery, no diabetes, and non-smoking. Fourteen healthy subjects (12 men, 2 women) participated in the main study and six subjects (3 men, 3 women) in the validation trial. Participants were asked to avoid intense physical activity and alcohol consumption the day before and during the test days. For the validation trial, they were additionally asked to reduce or avoid the consumption of C4 plant foods that have higher content of ^13^C (e.g., sorghum, millet, maize, and cane sugar-based products) during the testing period. Approval for the study was obtained from the Institutional Review Board of Purdue University (Protocol 1104010761, approved 24 May 2011) and the National Ethical Committee for Health and Life Sciences in Mali, and a written consent form was obtained from each subject before his or her participation in the study. The study is registered in clinicaltrials.gov (ID NCT03007368).

Subject impressions (*n* = 14) of differences between traditional and imported foods were assessed by a pre-test questionnaire following the completion of the consent form. Information gained from the questionnaire included subject food preferences, consumption frequency, and impressions of satiety effects of traditional and non-traditional staple foods.

### 2.2. Test Meals

Six solid foods were tested: white rice (long-grain, not parboiled), plain potato, wheat pasta, sorghum and millet thick (solid) porridges, and millet couscous. Two liquid foods were also tested: millet thin porridges (one plain and a second containing granules (*monikuru*) made from millet flour). Solid test meals were served with a tomato-based sauce made from onions, tomato paste, and fresh tomatoes, plus a seasoning of salt, black pepper, celery, and green pepper. The two thin porridges were served without addition of the sauce. All test meals were prepared on site based on typical preparation methods used in Mali. Rice, potatoes, and pasta were cooked to softness in slightly salted water. Thick porridges, and the plain thin porridge, were prepared from decorticated grains similar to the cooking methods described by Scheuring et al. [29]. For traditional thick porridge, approximately 4 L of water was boiled in a pot. Traditional potash (10 g) was mixed with approximately 650 mL cold water in a calabash or bowl, to which 750 g of flour (sorghum or millet) was added. The mixture was stirred until homogeneous and then swirled into the pot of boiling water. The boiling mixture was stirred for about 8 min to make a “bouillie” (thin porridge). The heat was then reduced, and about 1300 mL of the thin porridge was removed from the pot and set aside in another calabash. To the boiling thin porridge in the pot, 1250 g of sorghum (or millet) flour was added, one handful at a time. After each addition of flour, the paste was vigorously stirred with a flat wooden spoon. At different intervals of paste thickening, small amounts of thin porridge from the calabash were added. The process of flour addition followed by stirring and further addition of bouillie was continued for about 9 min until all the bouillie that had been set aside in the calabash had been used and the paste was thick and homogeneous. Then the fire under the pot was reduced and the thick paste was allowed to cook for about 12 min until done. For the thin porridge with granules, two-thirds of the flour was mixed with water to make the granules. The granules were then cooked in boiled water, and a slurry was made from the rest of the flour and added to the cooked granules. Lemon juice was added to the thin porridges for taste. Couscous was prepared by first mixing a decorticated coarse millet flour with a small amount of water and agglomerating it by hand to make small fine particles. Particles were then sieved with a medium diameter traditional sieve to obtain particle size uniformity, after which they were steamed in a couscoussier. Before adding the tomato sauce (250 g), ^13^C-octanoic acid (100 mg, Sigma-Aldrich, St. Louis, MO, USA) was thoroughly mixed into each meal portion (650 g) [30]. Test meals were allowed to sit for 10 to 20 min before being served (serving temperature of approximately 40–50 °C). Each subject was allowed to drink 150 mL of water with the meal.

### 2.3. Procedure

A crossover design study was performed in Bamako, Mali at the Sotuba Agricultural Research Center of the Institut d’Economie Rurale (IER) consisting of a main study (*n* = 14) and validation study (*n* = 6) (described below). On the morning of each test day, one of the test meals was prepared with addition of ^13^C-octanoic acid as a marker for assessment of gastric emptying rate [30,31,32,33]. All subjects consumed each type of test meal in the same randomly assigned order.

#### 2.3.1. Main Study (*n* = 14)

Eight test meals were given in replicate tests over 16 consecutive days, with all subjects provided the same meal on each day. Subjects were asked to arrive at the Sotuba Center at 9:30 a.m. and were instructed to fast overnight (>10 h) prior to the testing. Each test meal was given at 10:00 a.m. with instruction to consume as much as they would like until they felt full within a 15 min period [34]. Breath samples and subjective satiety ratings were collected from each subject in duplicate before consuming the test meal (baseline value), at 15 min intervals during the first 2 h, and then at 30 min intervals until 4 h. The test meals were weighed before and after they were presented to the subjects and from this the energy intake was calculated using food composition tables. The amount of test meal consumed on dry weight basis was converted into energy intake by multiplying the weight of the food by its caloric value [1,35]. Breath samples were analyzed using a ^13^C breath analyzer (POCone, Otsuka Co., Osaka, Japan), an infrared spectrophotometer that determines the ratio of ^13^CO_2_ to ^12^CO_2_ [36,37,38].

#### 2.3.2. Validation Study (*n* = 6) (Conducted Approximately One Year Later)

In the validation study, to account for the effect of intrinsic differences in background ^13^C, sorghum and millet porridges (thick and thin) and millet couscous were tested with and without added ^13^C-octanoic acid to provide baseline reference data for ^13^C in breath CO_2_ (associated with consumption of C4 sorghum and millet, which have higher amounts of endogenous ^13^C [39]). Rice, potatoes, and pasta (from C3 plants) were tested only with the added ^13^C-octanoic acid, because their endogenous ^13^C content was considered negligible. Otherwise, the same protocol was used as described above. The test meal size was 700 g.

#### 2.3.3. Consecutive-Day Testing of Baseline ^13^CO_2_ Values (*n* = 4)

Baseline breath ^13^CO_2_ content of each subject at zero time on each test day showed no buildup of baseline ^13^CO_2_ over a 6 consecutive-day testing period (Figure S1).

### 2.4. Calculation of Gastric Emptying Parameters

Raw data from the breath analyzer represents change in the ^13^CO_2_ DOB (delta over baseline, ‰), where the ^13^CO_2_/^12^CO_2_ ratio of a sample gas is compared to the corresponding ratio of a reference gas (i.e., baseline value). Using this value, CO_2_ production, percent dose ^13^C recovery per hour (PDR), and cumulative percentage dose recovery over time (CPDR) are calculated [36,38,40]. CO_2_ production was assumed to be 300 mmol/(m^2^ body surface area × hour), with body surface area calculated using the formula of Haycock et al. [41]. After calculating PDR and CPDR values, they were modeled as functions using the following equations to discern parameters related to gastric emptying:

y = at^b^c^−ct^, (1)

where y = percentage dose recovery per hour, t = time in hours, a, b, and c = constants; and

y = m(1 − e^−kt^)^β^, (2)

where y = cumulative percentage dose recovery over time, t = time in hours, m, k, and β = constants, and m = total cumulative dose recovery when time is infinite.

Modeling of data was achieved by nonlinear regression using SAS statistical analysis software (v.9.2, SAS Institute Inc., Cary, NC, USA) and was confirmed using a macro program in Excel (Microsoft Corp., Redmond, WA, USA). Gastric emptying parameters were then calculated using the following formulas: (1) lag phase (i.e., time required for the ^13^CO_2_ excretion rate to attain its maximal level) [42],

T(Lag) = (ln β)/k,
(3)

and (2) half-emptying time (i.e., time necessary for half of the ^13^C dose to be metabolized) [42],

(T_1/2_) = (−1/k) × ln (1 − 2^−1/β^). (4)

### 2.5. Satiety Questionnaire

In the main study, visual analogue scales (VAS, in mm) were used to assess fullness, hunger, desire to eat, and prospective food consumption [43], and were performed before eating the test meal, right after eating, and 2 and 4 h after test meal consumption. For example, fullness was rated by placing a mark on a 100 mm VAS at a position that showed degree of fullness between “Not at all full” on the left to “Extremely full” on the right. All other parameters (hunger, desire to eat, and prospective consumption) were rated in the same manner.

### 2.6. Statistical Analyses

The pre-test questionnaire results were reported as percentage of participants (*n* = 14) responding.

For comparison of the gastric emptying data of the main study, test foods were separated into solid and liquid groups. Solid foods were rice, boiled potatoes, pasta, sorghum and millet thick porridges, and millet couscous. Liquid foods were the two thin porridges. Comparisons of lag phase, half-emptying times, and values across foods, as well as energy intake values, were analyzed by one-way repeated-measure analysis of variance (ANOVA) with post-hoc Tukey tests used to form statistical groupings (α = 0.05). The amount of test meals consumed were reported as the mean value ± SEM and compared at α = 0.05 using one-way ANOVA with post hoc Tukey’s honest significant difference (HSD) test for multiple comparisons using IBM SPSS Statistics v.21 software package (version 21, IBM Corporation, Armonk, NY, USA).

Fullness, hunger, desire to eat, and prospective consumption scores of the different test meals were also compared at α = 0.05 using repeated measures analysis of variance with post hoc Tukey’s HSD tests for pairwise comparisons. The different ratings at each time point were compared using one-way ANOVA with post-hoc Tukey’s HSD test to form statistical groupings (α = 0.05).

## 3. Results

### 3.1. Pre-Test Questionnaire

In Mali, solid foods (rice, boiled potatoes, pasta, sorghum and millet thick porridges, and millet couscous) are consumed mostly for lunch and/or dinner, while thin porridges with granules are typically eaten for breakfast. Questionnaire results from the main study (*n* = 14) revealed that 100% of the participants consumed thin porridge with granules once per day as breakfast, while thin porridge without granules was never eaten (Table 1). This differs from other West Africa Sahelian countries where plain thin porridges are commonly consumed. The majority of participants reported frequent consumption of thick porridge, rice, and couscous (43%, 43%, and 36% more than once per week, respectively). Fifty percent reported eating thick porridge only once per week.

Rice was preferred at lunch for 71% of the participants (Table 2). Thin porridge with granules or potatoes were preferred for breakfast (equal response of 43% of participants), while millet thick porridge was preferred for dinner by 36% of participants. In terms of perceived satiating properties, millet couscous and rice were rated highest in the pre-test questionnaire with values of 43% and 36%, respectively (Table 2).

### 3.2. Subject Characteristics

#### 3.2.1. Main Study (*n* = 14)

Fourteen healthy volunteers (12 men, 2 women) between the ages of 20–26 years old (mean ± standard deviation (SD), 22.8 ± 2.1) with a mean (±SD) BMI of 20.5 ± 1.3 kg/m^2^ were recruited by local advertisement in the area of the research center at Sotuba, a peri-urban section of Bamako where the study was conducted (Table 3).

#### 3.2.2. Validation Study (Conducted Approximately One Year Later)

Six healthy volunteers (three men, three women) between the ages of 20–28 years old (24.3 ± 3.3) with a mean BMI of 22.2 ± 1.5 kg/m^2^ were recruited by the same method and in the same location as above (Table 3).

### 3.3. Consumption Amount and Energy Intake

Subjects consumed the test meals to fullness and, in both studies, consumption amounts of most meals were similar (Table 4). Exceptions were: in the main study, sorghum thick porridge was lower than thin porridge without granules (*p* < 0.05); and, in the validation study, sorghum thick porridge was lower than boiled potato and both thin porridges (*p* < 0.05), and pasta and millet couscous were lower than the two thin porridges (*p* < 0.05).

When put on an energy intake basis, further differences appeared. For the main study, among the solid foods, mean energy intake was lower for boiled potato (571.5 kcal) compared to rice (839.9 kcal), pasta (845.9 kcal), sorghum thick porridge (815.5 kcal), millet thick porridge (1011.5 kcal), and millet couscous (880.8 kcal), which were not different from each other. For the validation study, mean energy intake for the solid foods was again lower for boiled potato (485.5 kcal) compared to rice (770.2 kcal), pasta (629.8 kcal), sorghum thick porridge (542.3 kcal), millet thick porridge (853.6 kcal), and millet couscous (623.6 kcal).

### 3.4. Gastric Emptying of the Different Test Meals

#### 3.4.1. Main Study

After ingestion of the labeled octanoic acid, traditional African foods (sorghum thick porridge, millet thick porridge, millet couscous) were characterized by a long lag period of mean rate of recovery of ^13^C in breath followed by a plateau over time, whereas the non-traditional foods (rice, potatoes, pasta) had a short lag period followed by a decrease of recovery to the baseline (not shown). The two thin porridges showed similar ^13^C label recovery rates.

**Figure 1:**
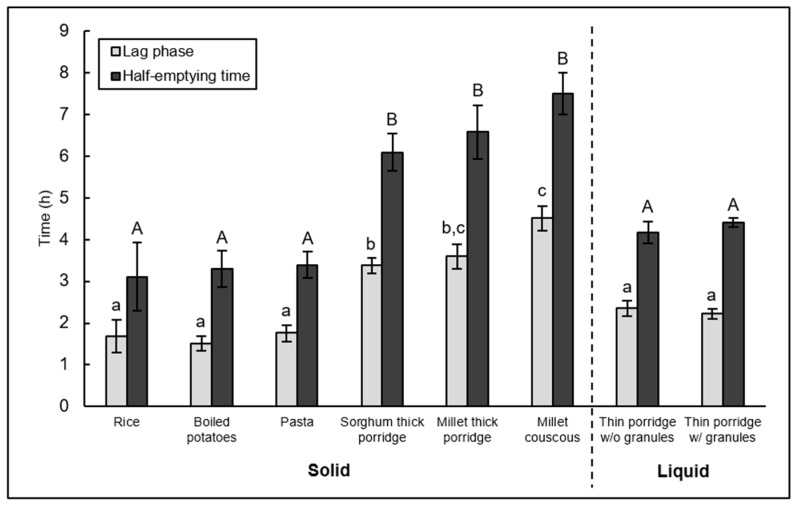
Gastric emptying parameters from the main study uncorrected for background differences in ^13^C content of food sources. Mean values (error bars (SEM), *n* = 14) of lag phase and half-emptying time of the different solid and liquid test meals. Means not sharing the same letter are significantly different (*p* < 0.05, statistical analysis was done separately for solid and liquid test meals). w/o = without, w/ = with.

Calculated values for gastric half-emptying (T_1/2_) and lag phase (T(Lag)) times showed an approximate doubling of each parameter in the traditional African solid foods compared to the non-traditional solid foods (*p* < 0.05, Table 5, Figure 1). Rice, boiled potatoes, and wheat pasta were faster emptying and did not differ significantly from one another for either parameter (*p* > 0.05). Slower emptying traditional foods (sorghum thick porridge, millet thick porridge, millet couscous) were the same for half-emptying time. The two thin millet porridges had gastric half-emptying and lag phase times comparable to the non-traditional solid foods. 

#### 3.4.2. Validation Study

Since the initial study did not take into account the higher endogenous ^13^C content in C4 plants (sorghum and millet) versus C3 plants (rice, potato, wheat), gastric emptying rate assessment was repeated using a smaller number of subjects (*n* = 6). Sorghum and millet thick and thin porridges and millet couscous were tested on two occasions with and without the addition of the ^13^C-octanoic acid. This provided baseline reference data for ^13^C in breath CO_2_ for ^13^C in the C4 plants. Rice, potato, and pasta were tested only with the ^13^C-octanoic acid, as their endogenous ^13^C contents are negligible. Supplementary Materials Table S1 shows the baseline data for postprandial ^13^CO_2_ after consumption of the sorghum and millet-based test meals. Baseline ^13^CO_2_ levels were found to increase somewhat. These values were subtracted, similar to a blank, from the same meals tested with the ^13^CO_2_-labeled octanoic acid. 

**Figure 2:**
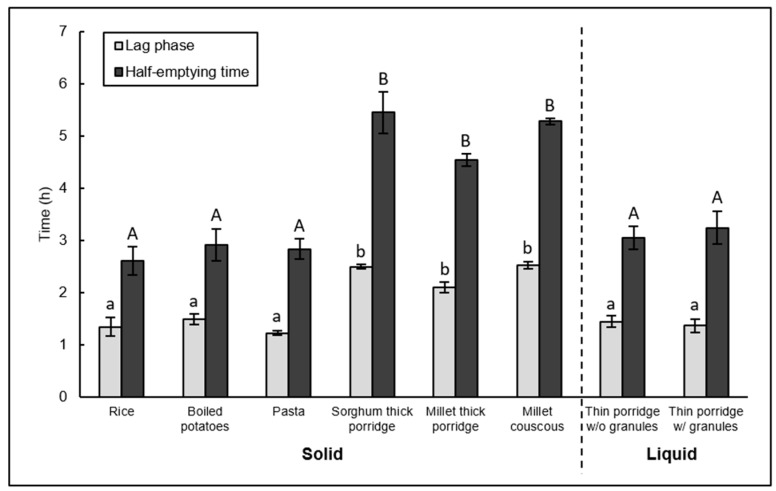
Gastric emptying parameters from the validation study, depicting correction for background differences in ^13^C content of food sources. Mean values (error bars (SEM), *n* = 6) of lag phase and half-emptying time of the different solid and liquid test meals. Means not sharing the same letter are significantly different (*p* < 0.05, statistical analysis was done separately for solid and liquid test meals). w/o = without, w/ = with.

Similar to the main study, corrected gastric half-emptying and lag phase times were about twice as high for the traditional African solid foods as the non-traditional solid foods (Table 5, Figure 2). Traditional sorghum and millet foods (sorghum thick porridge, millet thick porridge, and millet couscous) had slower gastric emptying compared to rice, potatoes, and pasta, and lag phase.

#### 3.4.3. Combined Study Results

Overall, the two studies conducted approximately a year apart, with the validation study corrected for baseline differences in endogenous ^13^C, showed remarkably similar results. The solid foods could be clustered into two distinct groups:

- Fast gastric emptying group: rice, boiled potatoes, and pasta
- Slow gastric emptying group: sorghum and millet thick porridges, and millet couscous

### 3.5. Satiety

**Figure 3:**
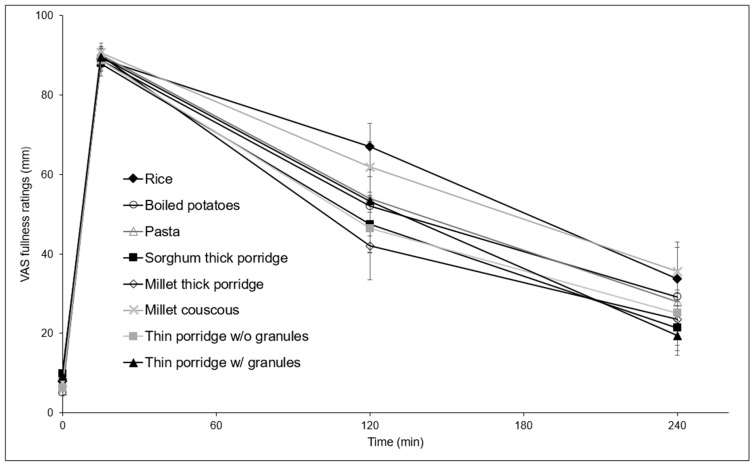
Mean subjective fullness ratings after ingestion of the different solid test meals (error bars (SEM), *n* = 14). VAS, visual analogue scales. w/o = without, w/ = with.

**Figure 4:**
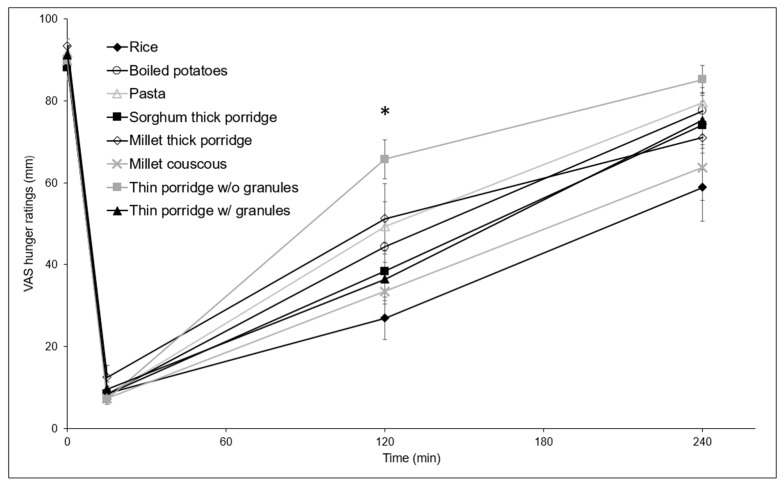
Mean subjective hunger ratings after ingestion of the different solid test meals (error bars (SEM), *n* = 14). * Statistically significant differences were found at 2 h between hunger ratings for thin porridge without granules and thin porridge with granules (*p* = 0.042), millet couscous (*p* = 0.016), and rice (*p* = 0.001). w/o = without, w/ = with.

Figure 3 and Figure 4 show mean fullness and hunger trends following consumption of the test meals. At 2 h, the hunger rating for thin porridge without granules was significantly higher than for thin porridge with granules (*p* = 0.042), millet couscous (*p* = 0.016), and rice (*p* = 0.001). Trends were similar for 2 and 4 h with millet couscous and rice segregating from others towards lower hunger and higher fullness, although there were no statistically significant differences among test meals at 4 h for hunger, or at 2 or 4 h for fullness. Statistically significant differences were found between fullness ratings at 2 and 4 h within test meal types for rice and thin porridge with granules (*p* < 0.05); and differences were found between hunger ratings at 2 and 4 h for rice, boiled potatoes, thin porridge with granules, and sorghum thick porridge (*p* < 0.05).

## 4. Discussion

The traditional solid African sorghum and millet foods (sorghum and millet thick porridges, millet couscous) had markedly slower gastric emptying rates compared to non-traditional foods (rice, potato, pasta) that are commonly consumed in urban Africa today. From the validation study, where endogenous ^13^C differences in food sources were corrected, gastric half-emptying mean values for the sorghum and millet foods were high (>50% of stomach contents were retained at 4 h; with 50% retention at 5.4 h for sorghum thick porridge, 4.5 h for millet thick porridge, and 5.3 h for millet couscous). Gastric half-emptying times were higher than the 4 h cutoff used by gastroenterologists to indicate abnormality in stomach emptying (severe delay in gastric emptying defined as >35% retention at 4 h) [44,45]. Apparently, though, this is normal for sorghum and millet traditional foods, at least for West Africans who normally consume these foods. There was not any indication of abnormality associated with this slow emptying rate, as is noted in the condition called idiopathic gastroparesis [45].

Although viscosity has been demonstrated in several studies to slow emptying rate [46,47,48], and this may explain the slow gastric emptying of the sorghum and millet thick porridges, the non-viscous millet couscous meal was also equally slow in emptying. This suggests that there is an intrinsic property of millet and sorghum grains that is associated with decreased gastric emptying rate. For the couscous, there could be a physical property of the particles that creates a movement through the stomach [49,50,51], or perhaps a slow digestion of the couscous particles that would trigger the ileal brake [47,52,53]. Indeed, sorghum and millet foods are known to have low starch digestibility, which may deposit glucose more distally in the small intestine [13,24,25].

Of the fast gastric emptying group, all presented low half-emptying and lag times. Perhaps puzzling is that pasta was fast emptying, as it has been reported to have relatively slow gastric emptying [54,55]. Likely, this is because the pasta in the study was well-cooked and the texture was soft, rather than al dente pasta that is harder in texture. 

Contents of fiber [56] and protein [57,58] in foods can also impact gastric emptying rate. However, in this study, the sorghum and millet grains used to make the traditional foods were decorticated, a debranning process where the majority of fiber is removed. Moreover, the majority of fiber in these grains is insoluble and does not contribute to viscosity, a factor shown to affect gastric emptying. As for protein, sorghum contains kafirin, which has been shown to relate to the slow starch digestion property of certain sorghum foods [13,59]; however, to our knowledge, no previous work has explored its effect on gastric emptying. Overall, there is no evidence that these factors resulted in the marked differences in gastric emptying rates observed in this study. 

In some studies, slow gastric emptying rates have been directly associated with low glycemic response [54,55], as well as satiation [18,20]. VAS scores on postprandial hunger at 2 h supported millet couscous as being more satiating, though also showed rice to be satiating. Because the pre-test questionnaire showed both to be more filling, perhaps preconceived notions of what foods are satiating may have influenced hunger and fullness scoring. Although protein has been found to increase satiation and satiety [60], small amounts show minimal effect [61] (our test meals were comparably low in protein). Fiber also has been found to have mixed effects on satiety [62], and likewise the amount of fiber in our test meals was low, as the grains were debranned. Therefore, it is unlikely that protein or fiber had an impact on the appetitive responses observed. Differences in energy density of the test meals could also have contributed to the gastric emptying rates and VAS scores observed [63,64]. Overall, the faster gastric emptying rates seen in the non-traditional foods tested (rice, potato, pasta) should increase glycemic response, as the starch component enters the duodenum for digestion at a faster rate.

## 5. Conclusions

Overall, traditional West African millet and sorghum thick porridges and millet couscous had substantially slower gastric emptying rates compared to non-traditional starchy foods (rice, potato, wheat pasta) that are consumed in African urban areas today. High gastric emptying times of greater than 4 h, with small variance around means, indicates that all subjects responded to the traditional foods in this way. In the West African context, slow gastric emptying would be considered as a food attribute connected with feelings of fullness and sustained energy.

As African populations have become urbanized, the change towards a more Westernized diet has been associated with rises in obesity and related metabolic syndrome diseases. The current study shows that in the West African Sahel, replacement starchy staple foods have fast gastric emptying compared to the slow gastric emptying of traditional sorghum and millet foods, and implies that the latter could be beneficial to health by providing energy from a meal over a longer time and imparting a satiety effect. Knowledge of this attribute of sorghum and millet foods could be useful to increase their consumption in West African cities and improve markets for local smallholder farmers.

## Figures and Tables

**Table 1:** Frequency (%) of consumption of the different test meals (*n* = 14).

Frequency of Consumption (%)	%						
	Rice	Boiled Potatoes	Pasta	Thick Porridge	Millet Couscous	Thin Porridge w/o Granules	Thin Porridge with Granules
>Once a day	28.6	0	0	0	0	0	0
Once a day	28.6	0	7.1	7.1	0	0	100
>Once a week	42.9	14.3	14.3	42.9	35.7	0	0
Once a week	0	21.4	21.4	50	28.6	0	0
>Once a month	0	21.4	28.6	0	7.1	0	0
Once a month	0	42.9	21.4	0	7.1	0	0
>Once a year	0	0	0	0	0	0	0
Once a year	0	0	7.1	0	21.4	0	0
Never eaten	0	0	0	0	0	100	0
Total	100	100	100	100	100	100	100

w/o = without.

**Table 2:** Frequency (%) of preference of the different test meals, and comment on fullness (*n* = 14).

Meal Type	%			
	Breakfast	Lunch	Dinner	More Filling
Rice	7.1	71.4	14.3	35.7
Boiled potatoes	42.9	0	21.4	7.1
Pasta	7.1	7.1	7.1	7.1
Sorghum thick porridge	0	0	0	0
Millet thick porridge	0	7.1	35.7	7.1
Millet couscous	0	14.3	14.3	42.9
Thin porridge w/o granules	0	0	7.1	0
Thin porridge with granules	42.9	0	0	0
Total	100	100	100	100

w/o = without.

**Table 3:** Subject characteristics.

Characteristics	Main Study (*n* = 14)	Validation Study (*n* = 6)
Age (years)	22.8 ± 2.1	24.3 ± 3.3
Height (cm)	175.7 ± 6.7	172.8 ± 12.4
Weight (kg)	63.4 ± 7.7	67.5 ± 10.6
BMI (kg/m^2^)	20.5 ± 1.7	22.5 ± 1.5

Values ± SD, standard deviation. BMI = body mass index.

**Table 4:** Amount of test meals consumed, wet weight (main study (*n* = 14); validation study (*n* = 6)).

Meal Type	Main Study Mean Amount Consumed, g	Validation Study Mean Amount Consumed, g
Rice	647 ^ab^ ± 27.3	575 ^abc^ ± 54.7
Boiled potatoes	661 ^ab^ ± 26.4	620 ^bc^ ± 50.4
Pasta	600 ^ab^ ± 17.9	446 ^ac^ ± 47.1
Sorghum thick porridge	642 ^a^ ± 28.9	427 ^a^ ± 36.5
Millet thick porridge	619 ^ab^ ± 35.1	522 ^abc^ ± 54.8
Millet couscous	656 ^ab^ ± 26.4	464 ^ac^ ± 39.8
Thin porridge with granules	755 ^ab^ ± 31.9	700 ^b^ ± 0.0
Thin porridge without granules	762 ^b^ ± 31.9	700 ^b^ ± 0.0

Values ± SEM, standard error of the mean. For a, b, and c, means not sharing the same letter are significantly different (*p* < 0.05). In the validation study, solid foods were consumed to fullness, though the two liquid thin porridges were totally consumed.

**Table 5:** Gastric half-emptying and lag phase times (main study (*n* = 14); validation study (*n* = 6)).

Meal Type	Main Study Mean T_1/2_ (h)	Validation Study Mean T_1/2_ (h)	Main Study Mean T(Lag) (h)	Validation Study Mean T(Lag) (h)
Rice	3.1 ^a^ ± 0.8	2.6 ^a^ ± 0.3	1.7 ^a^ ± 0.4	1.3 ^a^ ± 0.2
Boiled potatoes	3.3 ^a^ ± 0.4	2.9 ^a^ ± 0.3	1.5 ^a^ ± 0.2	1.5 ^a^ ± 0.1
Pasta	3.4 ^a^ ± 0.3	2.8 ^a^ ± 0.2	1.8 ^a^ ± 0.2	1.2 ^a^ ± 0.05
Sorghum thick porridge	6.1 ^b^ ± 0.4	5.4 ^b^ ± 0.4	3.4 ^b^ ± 0.2	2.5 ^b^ ± 0.04
Millet thick porridge	6.6 ^b^ ± 0.6	4.5 ^b^ ± 0.1	3.6 ^b^ ± 0.3	2.1 ^b^ ± 0.1
Millet couscous	7.5 ^b^ ± 0.5	5.3 ^b^ ± 0.1	4.5 ^b^ ± 0.3	2.5 ^b^ ± 0.1
Thin porridge with granules	4.2 ^a^ ± 0.3	3.0 ^a^ ± 0.2	2.4 ^a^ ± 0.2	1.4 ^a^ ± 0.1
Thin porridge without granules	4.4 ^a^ ± 0.1	3.2 ^a^ ± 0.3	2.2 ^a^ ± 0.1	1.4 ^a^ ± 0.1

Values ± SEM, standard error of the mean. For a, b, and c, Means not sharing the same letter are significantly different (*p* < 0.05, statistical analysis was done separately for solid and liquid test meals). T_1/2_ = gastric half-emptying time; T(Lag) = lag phase.

## Supplementary Materials

The following are available online at http://www.mdpi.com/2072-6643/10/2/124/s1, Figure S1: Average baseline ^13^CO_2_ values over 6 consecutive days (*n* = 4). Table S1: Validation study—mean values (*n* = 6) for postprandial ^13^CO_2_ associated with the higher endogenous ^13^C found in sorghum and millet-based test meals.
