# Supplementary material for: Traditional Malian Solid Foods Made from Sorghum and Millet Have Markedly Slower Gastric Emptying than Rice, Potato, or Pasta
Source: Nutrients. 2018 Jan 26;10(2):124. doi: 10.3390/nu10020124 (PMC5852700; doi:10.3390/nu10020124)
Supplement: Supplementary file 1 [file nutrients-10-00124-s001.docx]

Article – Online Supplementary Materials

Traditional Malian Solid Foods Made from Sorghum and Millet Have Markedly Slower Gastric Emptying than Rice, Potato, or Pasta

Fatimata Cisse ^1,2^, Daniel P. Erickson ^1,†^, Anna M. R. Hayes ^1^, Antone R. Opekun ^3^,
Buford L. Nichols ^3^ and Bruce R. Hamaker ^1,^*

^1^ Whistler Center for Carbohydrate Research, Department of Food Science, Purdue University, West Lafayette, IN 47907, USA; diallofati@gmail.com (F.C.); daniel.erickson@rd.nestle.com (D.P.E.); hayes100@purdue.edu (A.M.R.H.)

^2^ Institut d’Economie Rurale du Mali (IER), BP 258, Bamako, Mali

^3^ Departments of Pediatrics, Baylor College of Medicine, Houston, TX 77030, USA; aopekun@bcm.edu (A.R.O.); blnjr@sbcglobal.net (B.L.N.)

***** Correspondence: hamakerb@purdue.edu; Tel.: +765-494-5668

† Present address: Nestlé Purina Product Technology Center, 1 Checkerboard Square, Saint Louis, MO 63164, USA

Maximum DOB during testing

**Figure S1.** Average baseline ^13^CO_2_ values over 6 consecutive days (*n* = 4). Values depicted as delta over baseline (DOB, ‰), with Day 1 breath as the reference baseline. The dotted horizontal line represents general maximum DOB values observed during testing. Error bars represent standard error of the mean (SEM) among all subjects per day. No significant differences were evident across days (P > 0.05, one-way ANOVA).

**Table S1.** Validation study—mean values (*n* = 6) for postprandial ^13^CO_2_ associated with the higher endogenous ^13^C found in sorghum and millet-based test meals.^1^

| Time (min) | Sorghum Thick Porridge | Millet Thick Porridge | Millet Couscous | Thin Porridge without Granules | Thin Porridge with Granules |
| --- | --- | --- | --- | --- | --- |
| 0 | -0.1 | 0.0 | -0.1 | 0.0 | 0.0 |
| 15 | 0.3 | -0.4 | -0.2 | 0.3 | 0.4 |
| 30 | 0.8 | -0.1 | 0.2 | 0.6 | 0.7 |
| 45 | 0.9 | 0.1 | 0.2 | 1.4 | 1.3 |
| 60 | 1.1 | 0.4 | 0.6 | 2.0 | 1.9 |
| 75 | 1.5 | 0.8 | 0.9 | 2.4 | 2.5 |
| 90 | 2.0 | 1.2 | 0.9 | 2.8 | 2.8 |
| 105 | 1.8 | 1.7 | 1.6 | 3.3 | 3.3 |
| 120 | 1.9 | 2.2 | 2.3 | 3.5 | 4.0 |
| 150 | 2.9 | 2.6 | 2.8 | 3.6 | 4.7 |
| 180 | 2.8 | 3.0 | 2.9 | 3.3 | 4.7 |
| 210 | 3.3 | 3.4 | 3.2 | 3.2 | 4.8 |
| 240 | 3.3 | 3.3 | 3.5 | 3.0 | 5.2 |

^1^These were treated as baseline reference samples and values were subtracted from ^13^C-octanoic acid gastric emptying test values for these foods in the validation study (*n* = 6).

© 2018 by the authors. Licensee MDPI, Basel, Switzerland. This article is an open access article distributed under the terms and conditions of the Creative Commons Attribution (CC BY) license (http://creativecommons.org/licenses/by/4.0/).
